# Case report of belt electrode-skeletal muscle electrical stimulation for acute heart failure with severe obesity: a novel therapeutic option for acute phase rehabilitation

**DOI:** 10.3389/fcvm.2024.1344137

**Published:** 2024-03-08

**Authors:** Yuto Mochizuki, Takahiro Jimba, Syota Yasukawa, Aritomo Katsura, Akira Fukuda, Jiro Ando

**Affiliations:** ^1^ Department of Rehabilitation Medicine, NTT Medical Center Tokyo, Tokyo, Japan; ^2^ Department of Cardiovascular Medicine, Graduate School of Medicine and Faculty of Medicine, The University of Tokyo, Tokyo, Japan; ^3^ Department of Cardiovascular Medicine, NTT Medical Center Tokyo, Tokyo, Japan

**Keywords:** cardiac rehabilitation, B-SES, NMES, heart failure, obesity, skeletal muscle

## Abstract

**Background:**

Belt electrode skeletal muscle electrical stimulation (B-SES) is an emerging therapy anticipated to yield more favorable outcomes than conventional neuromuscular electrical stimulation (NMES), owing to its larger stimulation area. However, information on its efficacy and safety in patients with heart failure remains limited.

**Case presentation:**

A 43-year-old man with a body mass index of 41 kg/m^2^ was admitted to our hospital for acute heart failure due to dilated cardiomyopathy. The patient required prolonged catecholamine support owing to poor cardiac function, and heart transplantation was considered. We initiated a mobilization program, but the patient's mobility was highly limited due to severe obesity and symptomatic orthostatic hypotension. B-SES was introduced to accomplish weight loss and early ambulation. We applied an intensive monitoring program for safe use and modulated the intensity of B-SES according to physical function. During the B-SES program, the patient's body weight decreased from 89.6 kg to 78.6 kg. Sequential evaluations of body composition and skeletal muscle ultrasonography revealed improved muscle mass, quality, and physical function. Furthermore, we explored the workload of B-SES using expiratory gas analysis. No adverse events were observed during B-SES.

**Discussion:**

We successfully used B-SES to improve muscle function and morbidity in the treatment of acute heart failure. B-SES could be an option for patients with heart failure who have limited mobility and obesity.

## 1 Introduction

Skeletal muscle dysfunction is common in patients with heart failure (HF) and is considered a dominant cause of easy fatigability and exercise intolerance. Deconditioning contributes to skeletal muscle wasting, which is particularly important in the treatment of patients with acute heart failure (AHF). Early mobilization and exercise therapy are important in preventing muscle abnormalities due to deconditioning (1). However, physical activity is often restricted by unstable circulatory dynamics in patients with AHF, and the implementation of acute-phase rehabilitation for these patients presents considerable challenges.

Neuromuscular electrical stimulation (NMES) is an alternative exercise therapy. NMES has been reported to be safe and feasible for patients with AHF (2). Recently, belt electrode skeletal muscle electrical stimulation (B-SES) has been introduced. The B-SES is set around the lower trunk, thighs, and ankles, and has a larger stimulation area than traditional NMES devices. Expanding the electrode area enables pain reduction during stimulation (3), which contributes to high-intensity muscle contraction in both lower limbs. B-SES has been reported to improve muscle mass and strength in patients on hemodialysis (4). However, an increased amount of muscle stimulation could alter circulatory dynamics, and its safety and efficacy in patients with AHF remain elusive. Herein, we report a case of successful B-SES in a patient with AHF and severe obesity. The patient's mobility was highly limited owing to unstable circulatory and overweight status. B-SES can be safely performed with intensive monitoring during catecholamine support. Based on the sequential evaluation of body composition and skeletal muscle ultrasonography, we showed that B-SES enabled weight loss and improved muscle function in a patient with HF who had limited mobility and was unresponsive to traditional cardiac rehabilitation.

## 2 Case description

A 43-year-old man with HF secondary to dyspnea was admitted to our hospital. The patient's height was 160 cm, body weight was 109 kg, and body mass index was 42.5 kg/m^2^. Past medical history included diabetes mellitus and sleep apnea. Upon admission, the patient experienced orthopnea and whole-body edema. Laboratory data indicated a creatinine of 1.3 mg/dl, an estimated glomerular filtration rate of 49 ml/min/1.73 m^2^ and an N-terminal pro-brain natriuretic peptide level of 4,177 pg/ml. Electrocardiography revealed sinus tachycardia and a left ventricular ejection fraction of 20% with diffuse left ventricular hypokinesis. Plain chest radiography revealed a cardiothoracic ratio of 66% and a costophrenic obtuse angle. The patient was diagnosed with AHF and was treated with intravenous furosemide and oxygen inhalation. However, the patient developed respiratory failure and low-output syndrome on the 7th hospital day. The patient was transferred to the cardiac care unit (CCU), and treatment with noradrenaline, dobutamine, and noninvasive positive pressure ventilation was initiated. On the 19th hospital day, right heart catheterization indicated Forrester subset IV (pulmonary artery wedge pressure, 27 mmHg; cardiac index: 1.6 L/min/m^2^), and coronary angiography revealed no significant stenosis. Based on the clinical course and endomyocardial biopsy of the right ventricular septum, the patient was diagnosed with idiopathic dilated cardiomyopathy on the 23rd hospital day. Because the patient was young and had developed catecholamine-dependent HF, heart transplantation was considered. However, severe obesity was an obstacle to registration. We started B-SES (G-TES; Homer Ion Laboratory, Tokyo, Japan) in parallel with normal cardiac rehabilitation and performed gradual withdrawal of catecholamines and titration of bisoprolol. On the 48th day of hospitalization, the patient was withdrawn from noradrenaline. The patient was transferred from the CCU to the general ward on the 92nd day. The patient's symptoms improved to NYHA II with titration of bisoprolol, ivabradine, valsartan, spironolactone, dapagliflozin and furosemide, along with cardiac rehabilitation. In addition, we provided life modification program to the patient because eating habits and sedentary life style had contributed to his obesity. The patient was discharged with a body weight of 75.6 kg on the 113th hospital day. This case report has anonymized patient information, and photographs including parts of the body (including the face) have been processed to ensure that the individual cannot be identified. We obtained written informed consent from the patient for publication.

## 3 Diagnostic assessment

**Figure 1:**
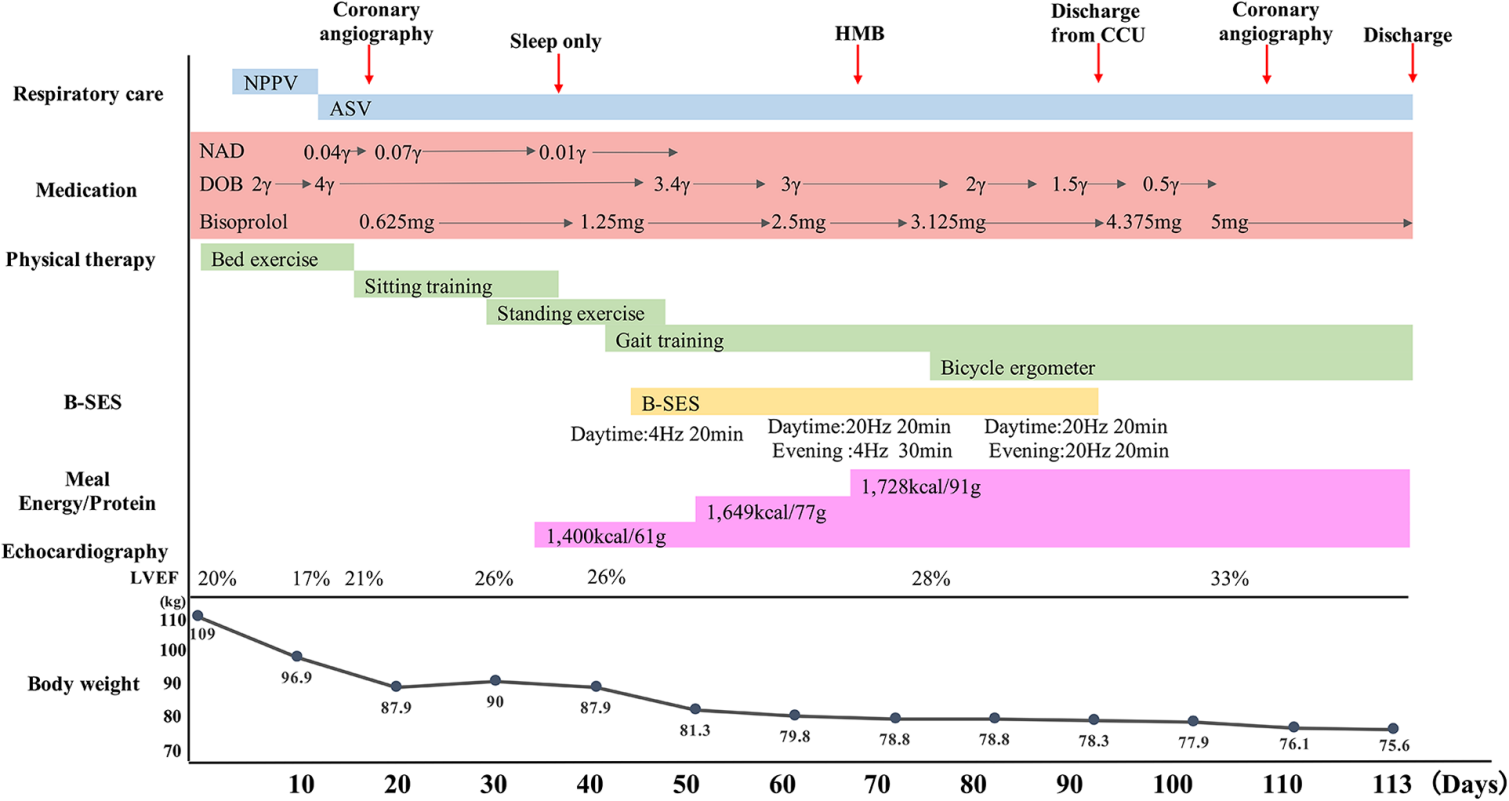
Clinical course. Respiratory care, medication, physical therapy, B-SES, energy/protein, LVEF and body weight are shown as a time course. ASV, adaptive servo ventilation; B-SES, belt electrode-skeletal muscle electrical stimulation; DOB, dobutamine; HMB, βhydroxy-βmethylbutyrate; LVEF, left ventricular ejection fraction; NAD, noradrenalin; NPPV, Non-invasive positive pressure ventilation.

The clinical course and contents of the rehabilitation program are shown in Figure 1. Range of motion and active assistive movement were performed from the 2nd to the 15th day due to unstable circulatory dynamics. Although sitting exercises were initiated on the 16th day, orthostatic hypotension and subjective fatigue were still observed. Standing exercises were initiated on the 30th day; however, the patient was unable to stand up because of orthostatic hypotension and leg fatigue. On the 43rd day, gait training in the room was initiated using a walker, and physical activity was interrupted due to orthostatic hypotension and subsequent fatigue.

**Figure 2:**
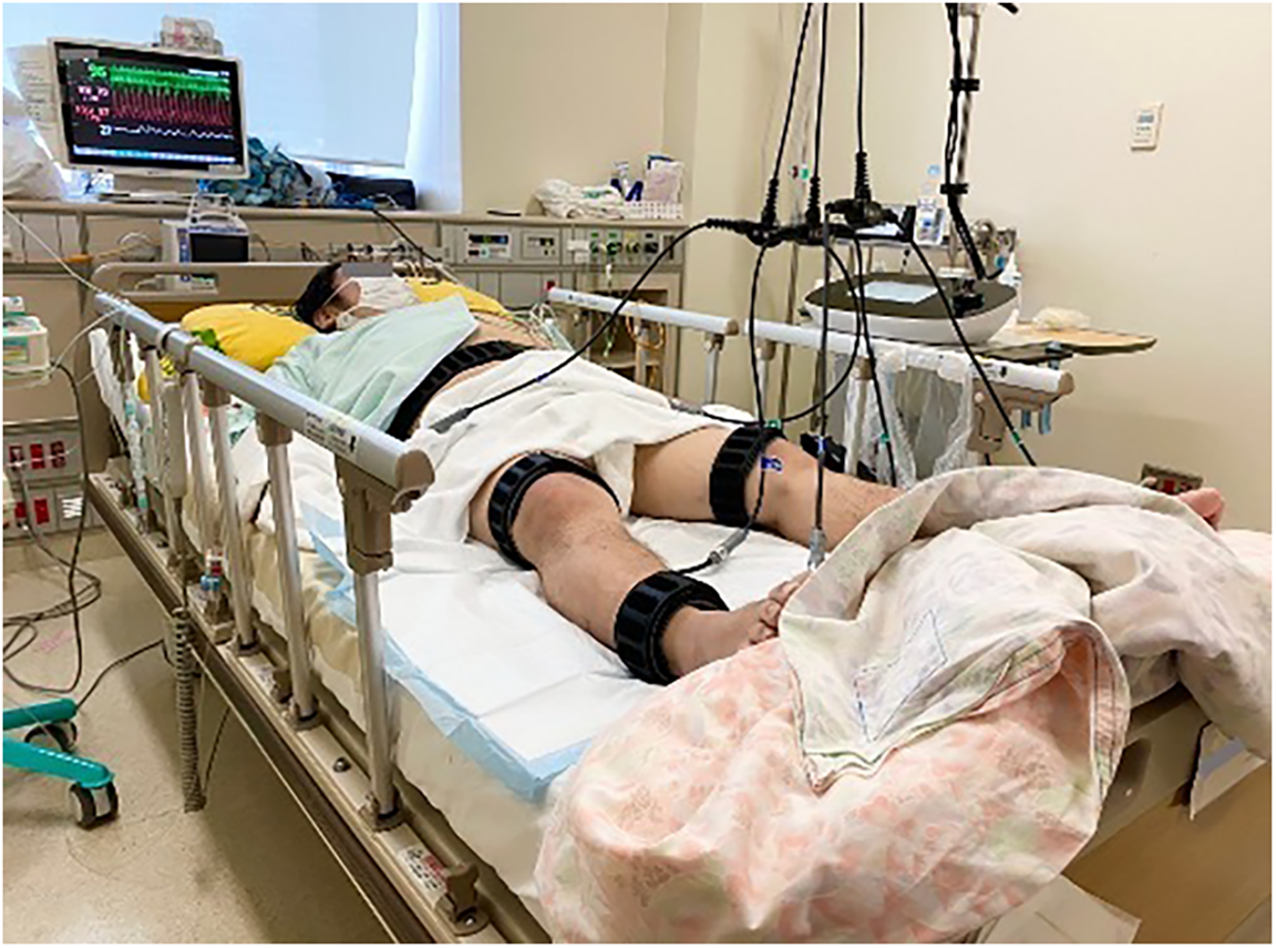
Belt electrode-skeletal muscle electrical stimulation. Position was head-up to 10°.

Weight loss has been identified as a major issue in improving mobility and registration for heart transplantation. In addition to normal cardiac rehabilitation, B-SES was started on the 48th day. The B-SES was placed around the lower trunk, thigh, and ankle (Figure 2). All muscles were simultaneously contracted for 20 min by an exponential growth wave of 250 ms pulses at a frequency of 4 Hz (5). At first, based on supplier recommendation and previous research, we have adopted low intensity protocol with a frequency of 4 Hz (6). The stimulus intensities were set to could cause visible contractions (7) and increased to the maximum intensity tolerated by the patient within 3 days. The patient demonstrated good tolerance to B-SES, allowing for active participation, and B-SES was performed daily for 6 weeks. Heart rate (HR), blood pressure, double product (DP), and Borg scale scores were evaluated at 5, 10, 15, and 20 min, respectively, after the sessions started (8). At first, B-SES was performed in the supine position; however, systolic blood pressure (SBP) and DP increased. We changed the position to head-up at 10°, which improved treatment tolerability. In addition, we assessed the creatine kinase and glomerular filtration rate as the marker of overload, and no excessive elevation of creatine kinase and no decrease in glomerular filtration rate were observed during the B-SES period.

We evaluated the patient's muscle strength, quality, and physical performance (Table 1). Body composition was measured by using a bioelectrical impedance device (Body Composition Analyzer, InBody S10; InBody Japan Co., Ltd., Tokyo, Japan). Leg muscle mass, thigh circumference, and lower leg circumference were obtained as the averages of the left and right sides. Transverse images of the rectus femoris (RF) of the right thigh were obtained using real-time B-mode ultrasonography (Vivid S6; GE Healthcare, Tokyo, Japan). Ultrasonography was performed to examine muscle thickness (MT) and subcutaneous fat thickness (FT). In addition, muscle quality of the lower limbs was assessed using echo intensity (EI) images obtained from ultrasonography. Images of FT, MT of the RF (RF_MT_), and EI of the RF (RF_EI_) were obtained halfway between the anterior superior iliac spine and the proximal end of the patella (9). Physical function was evaluated every 2 weeks, and the treatment of B-SES were consequently modified. In this case, ultrasonography in the 2nd week revealed a decrease in FT, but no improvement was observed in RF_MT_ and RF_EI_. Consequently, the settings of the B-SES were upregulated to a stimulus of 20 min at a frequency of 20 Hz, in the daytime, and a stimulus of 30 min in the evening at a frequency of 4 Hz. For high intensity protocol, we have adopted a frequency of 20 Hz, as per the effective frequency proposed in the past report (10).

**Table 1:** Changes in clinical parameter from start to end of B-SES.

	At start (46 days)	2nd week (60 days)	4th week (74 days)	6th week (88 days)
Muscle strength				
10RM the knee extensor (kg)	2	5	8	10
Grip strength (kg)		26.2	26.9	28.7
6 m walking speed (sec)		8.87	7.17	7.30
Total walk distance (m)	20	160	450	750
Body composition				
Body weight (kg)	87.9	79.8	79.0	78.6
Body fat mass (kg)	39.8	37.6	34.5	32.4
Muscle mass (kg)	47.4	39.9	42.1	43.7
Leg muscle mass (kg)	7.88	6.16	6.75	7.26
SMI (kg)	8.5	5.3	5.7	7.6
ECW/TBW	0.400	0.385	0.385	0.394
Waist circumference (cm)	121	106	105	104.2
Thigh circumference (cm)	52.1	48.7	47.3	48.0
Lower leg circumference (cm)	39	37	37.1	38.1
Ultrasound measurement				
FT (mm)	16.3	15.3	13.8	13.2
RF_MT_ (mm)	13.4	13.6	14.4	18.6
RF_EI_ (pixel)	64.4	60.0	26.4	40.6

ECW/TBW, extracellular water/total body water; FT, subcutaneous fat thickness; RF_EI_, rectus femoris echo intensity; RF_MT_, rectus femoris muscle thickness; RM, repetition maximum; SMI, skeletal muscle mass index.

In addition to using B-SES, nutrition therapy was planned with an energy requirement of 1,408–1,689 kcal and an amount of protein of 1.2–1.5 g/kg/day. Two medimeals® Leucine plus and one protein jelly were added as nutritional supplementary food, and one medimeal® Leucine plus was taken before starting B-SES to reduce fatigue during exercise and suppress muscle proteolysis (11). On the 50th day, gait training in the corridor was initiated with a walker. According to the up-regulation of the settings of B-SES and the contents of the trainings, nutritional therapy was changed to an energy requirement of 1,400–1,800 kcal. In addition, βhydroxy-βmethylbutyrate was ingested before and after B-SES. On the 75th day, aerobic exercise was initiated with Strength Ergo (Strength Ergo5 BK-ERG-051; Mitsubishi Mitsui Engineering Corporation, Aichi, Japan) in the rehabilitation room. Both daytime and evening B-SES settings were changed to a frequency of 20 Hz and stimulation time of 20 min on the 77th day. On the 92nd day, B-SES used ended due to discharge from the CCU, and gait training in the ward was included as a voluntary exercise. On the 113th day, the patient was discharged from the hospital.

**Figure 3:**
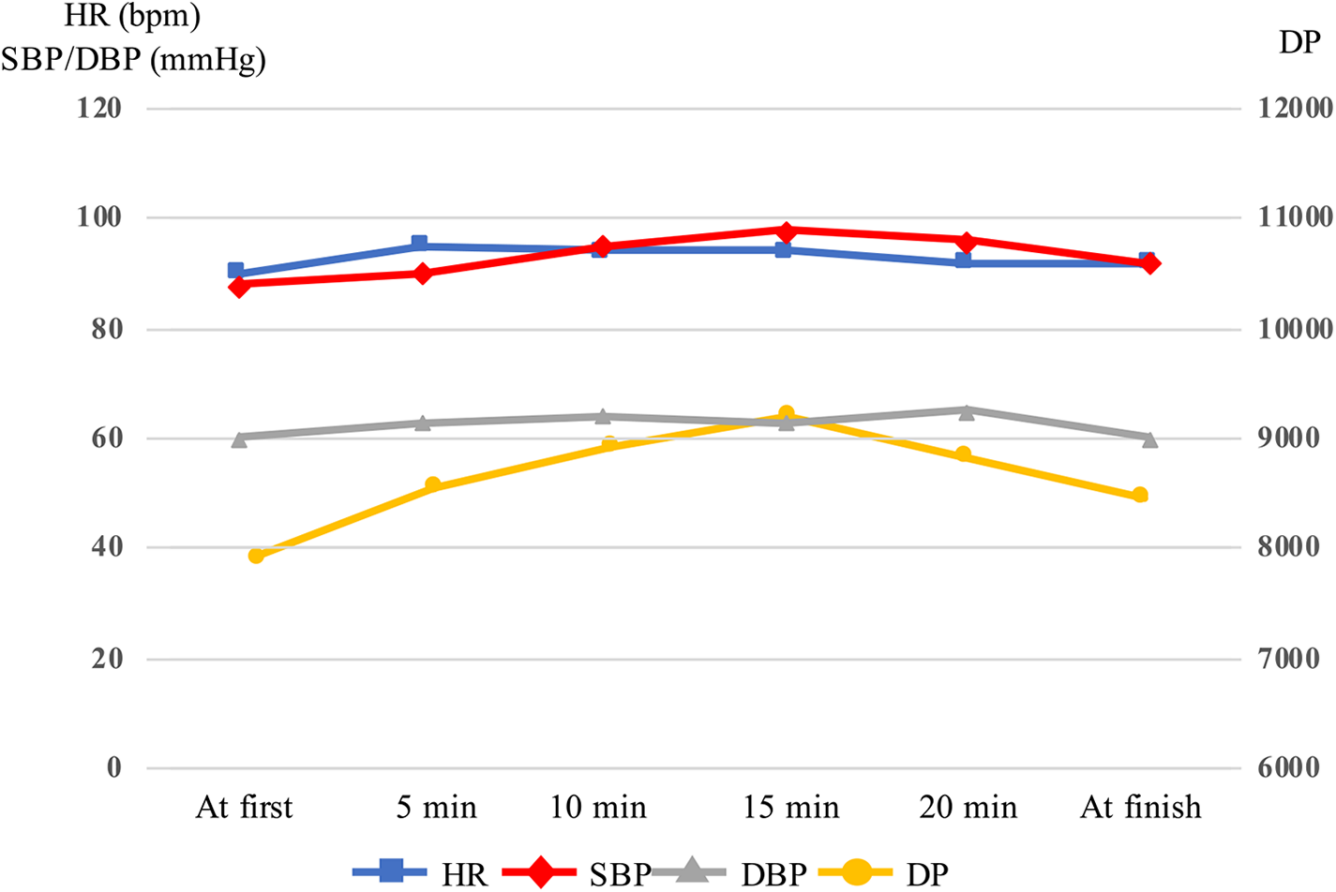
Variations in heart rate, systolic blood pressure, diastolic blood pressure and double product in belt electrode-skeletal muscle electrical stimulation. The head was raised to 10° 15 min after starting, and SBP and DP was decreased. DBP, diastolic blood pressure; DP, double product; HR, heart rate; SBP, systolic blood pressure.

The results of the physical function assessments are summarized in Table 1. Muscle strength and physical performance improved gradually. Body composition analysis revealed that body weight and fat mass had decreased, whereas muscle mass had increased. Muscle ultrasonography demonstrated that the FT had gradually decreased. The RF_MT_ and the RF_EI_ improved after up-regulation of the settings of the B-SES at the 2nd week. No adverse events were observed using B-SES. HR, SBP, and DP continued to increase after starting B-SES but decreased after 15 min (Figure 3).

We performed an expiratory gas analysis to estimate the exercise load and calorie expenditure during B-SES. Volume of oxygen (VO_2_) uptake was measured using an expired gas analyzer (Aeromonitor AE-310S; Minato Medical Science, Osaka, Japan) in the resting position and during B-SES. The B-SES settings included a frequency of 20 Hz and a stimulus of 20 min. Gas samples were collected for 5 min or more, and the average VO_2_ after breathing stabilization was analyzed. Calorie expenditure during B-SES was calculated using the formula: kcal/min = 3.5 × METs × body weight (kg)/200 (12). This expiratory gas analysis revealed that VO_2_ in the resting lying position was 293.1 ± 34.9 ml and VO_2_ during B-SES was 455.9 ± 15.6 ml. Calorie expenditure during B-SES was 65.5 kcal.

## 4 Discussion

To our knowledge, this is the first report to demonstrate the effective and safe application of B-SES in patients with severe cardiac dysfunction. Using B-SES, we improved skeletal muscle function and facilitated weight reduction in a patient who had difficulty ambulating due to severe HF and obesity.

NMES has been shown to attenuate proteolysis and prevent muscle atrophy in critically ill patients (13). In addition, NMES has the effects of aerobic exercise and resistance training (14), which have been reported to be amplified by increased loading (15). B-SES can induce high-intensity muscle contraction throughout both lower limbs owing to its large electrode area and offers a higher exercise potential than conventional NMES. The weekly duration of treatment has been reported to be associated with effectiveness, with a recommendation of at least 25 min per day (10). In consideration of the low cardiac function we have combined both low intensity and high intensity protocol, extending the daily duration to a maximum of 50 min. In the present case, we observed a substantial improvement in the quality and quantity of skeletal muscles, as demonstrated by body composition analysis and skeletal muscle ultrasonography, during B-SES. In addition, we combined B-SES with nutritional therapy using βhydroxy-βmethylbutyrate, which has been shown to have anabolic effects on skeletal muscles (16). This combined strategy could enhance the exercise effect of B-SES.

In the present case, body weight significantly reduced after using B-SES. From the body composition analysis, extracellular water/total body water (ECW/TBW) was high at the 1st week and decreased in the 2nd week along with the improvement of the sign of systemic congestion. This observation indicates that weight loss during this period was due to the reduction of body fluid volume by diuretics. After the 2nd week, body fat mass decreased, and muscle mass increased over time, whereas ECW/TBW was in normal range and did not change. This result indicated that the reduction in body fat mass could be attributed to weight loss in this phase. The short-term effect of NMES on body composition has been under investigation. A previous study (17), applying NMES to patients with sarcopenia for 4 weeks, reported a significant reduction in fat mass after introduction of NMES. In this case, the increase in the intensity of acute phase physical rehabilitation after the initiation of B-SES could also contribute to decrease in body fat mass. Patients with HF often present with comorbid obesity, which is associated with reduced exercise capacity and poor prognosis (18). However, weight reduction has been related to reverse and favorable cardiac remodeling and improved vascular function (19, 20). In this case, orthostatic hypotension and leg fatigue were attenuated after the introduction of B-SES, and the total walk distance gradually increased. Weight loss and improvement in lower skeletal muscle function could increase the venous return volume when standing, which might contribute to the attenuation of orthostatic hypotension.

Expiratory gas analysis demonstrated that the calorie expenditure during B-SES for 20 min was 65.5 kcal. In this case, 6-week sessions of B-SES led to a total expenditure of approximately up to 4,585 kcal. Body composition evaluation revealed a decreased body fat mass of 7.4 kg. This might not only be due to the direct exercise effect of B-SES but also to improved muscle function and subsequent improvement in basal metabolism and physical activity. Further studies should investigate the beneficial effect of B-SES with an expanded sample size and a well-defined control group.

Although B-SES has potential beneficial effects, safety concerns arise, especially when applied to vulnerable patients with severe cardiac dysfunction. In previous studies, blood pressure, HR, and creatine phosphokinase levels did not vary during conventional NMES in patients with severe congestive HF (NYHA Ⅲ–Ⅳ) (21). However, compared with NMES, the larger stimulation area of B-SES could have a greater effect on hemodynamics. Intensive hemodynamic monitoring was performed during the procedure. Indeed, in our first session, the SBP and DP continued to increase after starting B-SES and did not reach a stable state in the supine position. Venous return volume is increased by skeletal muscle contraction, leading to an increase in cardiac preload. In the present case, SBP and DP decreased when the head was raised to 10°, and the circulatory state was stabilized. Based on the expiratory gas analysis, the exercise intensity of B-SES was estimated to be 1.6 METs. B-SES can be safely applied to patients with AHF through careful patient selection, ensuring tolerance to this exercise intensity, and implementing proper management of pre-load under intensive monitoring. Nevertheless, further studies should investigate the optimal mode of NMES, including whole-body electrical muscle stimulation, as well as the appropriate method of patient selection and monitoring for AHF patients.

Through longitudinal evaluation of the patient, quantitative, qualitative, and functional improvements in the muscles were observed during B-SES. Furthermore, intensive monitoring and modification of the position during the procedure should be considered for safe application. B-SES may be a novel therapeutic option for patients with AHF, especially those with obesity who are unresponsive to conventional acute-phase rehabilitation.

## Data Availability

The original contributions presented in the study are included in the article/Supplementary Material, further inquiries can be directed to the corresponding author.
